# Intergroup encounters in Verreaux’s sifakas (*Propithecus verreauxi*): who fights and why?

**DOI:** 10.1007/s00265-016-2105-3

**Published:** 2016-03-30

**Authors:** Flávia Koch, Johannes Signer, Peter M. Kappeler, Claudia Fichtel

**Affiliations:** Behavioral Ecology and Sociobiology Unit, German Primate Center, 37077 Göttingen, Germany; Department of Wildlife Science, University of Göttingen, Büsgenweg 3, Göttingen, 37077 Germany; Department of Sociobiology and Anthropology, University of Göttingen, 37077 Göttingen, Germany

**Keywords:** Intergroup conflict, Participation, Free-riding, Collective action problem, Verreaux’s sifakas

## Abstract

**Abstract:**

Individuals living in groups have to achieve collective action for successful territorial defense. Because conflicts between neighboring groups always involve risks and costs, individuals must base their decision to participate in a given conflict on an evaluation of the trade-off between potential costs and benefits. Since group members may differ in motivation to engage in group encounters, they exhibit different levels of participation in conflicts. In this study, we investigated factors influencing participation in intergroup encounters in Verreaux’s sifakas (*Propithecus verreauxi*), a group-living primate from Madagascar. Over a period of 12 months, we studied eight adjacent sifaka groups in Kirindy Forest. We observed 71 encounters between known neighboring groups in which adult females and males participated equally as often. No individual participated in every encounter, and non-participation occurred more often in larger groups. Females participated less often in encounters when they had dependent infants, presumably to reduce the risk of infanticide. Male participation was influenced by social status: dominant males participated in most encounters, whereas males with fewer opportunities to reproduce participated less often, hence male participation is influenced by the incentive of maintaining access to females. The number of actively participating individuals in the opponent group positively influenced the participation in both sexes. Thus, sifakas seem to decide joining a given encounter opportunistically, most likely based on a combination of individual incentives and the actual circumstance of each encounter, suggesting that the complexity in intergroup relationships appears to be the product of decisions made by each individual group member.

**Significance statement:**

Cooperation among group-living animals is often challenged by collective action problems resulting from individual differences in interests in contributing to collective behaviors. Intergroup encounters involve distinguished costs and benefits for each individual despite being in the same social group. Therefore, encounters between groups offer a good opportunity to investigate individual participation in collective action. In this study, we investigate the influence of different incentives on individual participation in intergroup encounters in wild Malagasy primate, Verreaux’s sifakas. We propose a novel approach that takes into account the variable circumstances of each conflict, such as the number of individuals fighting in both groups as a predictor for participation. We believe that our study not only provides novel data on wild sifakas, but it also offers new perspectives for the interpretation of intergroup relationships in other taxa.

## Introduction

Dyadic conflicts over various resources are common in most animal taxa (Riechert 1979; Rood 1986; McComb et al. 1994; Crofoot and Wrangham 2010; Doake and Elwood 2011). Potential benefits from dyadic conflicts include access to valuable resources, such as food and/ or mates (Fashing 2001; Crofoot and Wrangham 2010). However, conflicts always involve some risks and costs, including physical aggression that may result in injury or even death (Williams et al. 2004; Kelly 2005). Thus, animals must base their decision to engage in a given conflict on an evaluation of the trade-off between potential costs and benefits (Parker 1974). Selection should therefore have promoted cognitive and behavioral strategies that enhance the ability of individuals in most non-sessile species to assess the value of a disputed resource, their own fighting ability, and the fighting ability of their opponent in order to estimate their chances of winning (reviewed in Arnott and Elwood 2008).

Whereas much theoretical and empirical research has examined dyadic conflicts between individuals (Landau 1951; Parker 1974; Dugatkin 1998), scramble and/or contest competition also occurs between groups in gregarious species. However, patterns and strategies characterizing intergroup conflict remain poorly understood; perhaps because they exhibit much more complex dynamics. For example, individuals in a group differ in intrinsic traits (size, physiological condition, age, rank, sex, motivation, personality) as well as in prior experience of winning and losing that determine their current fighting ability and their willingness in participating in group encounters (Olson 1965; Heinsohn and Packer 1995; Nunn and Deaner 2004; Harris 2010). Differences in these characteristics and interests among individuals in the same social group can influence their participation in group encounters, which may result in collective action problems (Hardin 1968; Rankin and Kokko 2007). Free-riding is indeed a common collective action problem observed in between-group conflicts (Olson 1965; Esteban and Ray 2001; Willems et al. 2013), which can decrease individual motivation in participating in a conflict (Nunn 2000). The outcome of decision-making at the group level is difficult to predict because it reflects the result of multiple, perhaps inter-dependent individual assessments of the balance between these costs and benefits. However, the observed outcome, i.e., which individuals participate in intergroup conflict, can be easily observed and analyzed in analogy to an individual decision process (Esteban and Ray 2001; Sumpter 2006; Crofoot et al. 2008).

There appear to be two important determinants of variation in individual participation in group encounters that we define as follows: the *incentive* reflects individual motivation, interest, or potential benefits (such as immediate access to a contested resource or access to mating partners) that an individual expects from an encounter, whereas the *circumstance* characterizes the general characteristics of a given encounter, such as the size and identity of the opponent group, the duration of the encounter, the presence of infants (avoidance of infanticide), and/or general food availability. For instance, the individual incentives to participate in a group encounter in mammals are expected to be strongly predicted by sex because the fitness of males is limited by access to mates, whereas the fitness of females is limited by access to food (Trivers 1972). As a result of this fundamental sex difference, males tend to have higher average incentives to participate in intergroup encounters more often than females; a pattern that has indeed been established empirically (Perry 1996; Fashing 2001; Sicotte and Macintosh 2004; Williams et al. 2004; Kitchen and Beehner 2007; Mares et al. 2012; Willems et al. 2013).

Moreover, high-ranking group members may monopolize a disproportionate share of the immediately available benefits, which may increase their incentive to participate in group encounters, compared with low-ranking group mates (Janson 1985; Nunn 2000; Kitchen et al. 2004; Cooper et al. 2004; Majolo et al. 2005). For instance, in species with pronounced male reproductive skew, dominant males may have stronger incentives to participate in group encounters because they have priority of access to mates (Cooper et al. 2004). In chimpanzees (*Pan troglodytes*), for example, the participation of males in border patrols increases according to the benefits those males can expect from the conflict (Watts and Mitani 2001). Thus, individual incentives vary, and they may do so for very different reasons (low prospects and free-riding generally predict no or rare participation).

The effects of the particular circumstances of an intergroup encounter on the probability of individual participation remain less well understood, however. The size and power of the opposing group can influence individual participation in group encounters because it factors into the assessment of the costs of a conflict (Parker 1974). For example, individual participation of female lions (*Panthera leo*) in territorial disputes increases if they have a numerical advantage over the other group (McComb et al. 1994). The importance of differences in group size is reflected by the observation that large groups tend to indeed defeat smaller ones (Black and Owen 1989; Holldobler and Wilson 1990; McComb et al. 1994; Wilson et al. 2001; Kitchen et al. 2004; Crofoot et al. 2008; Brown 2011; Cassidy et al. 2015). The duration of encounters can also influence individual participation. Since long encounters are physically more demanding than shorter ones, due to the tendency of increased levels of aggression, it can be predicted that the number of participants increases with the duration of the encounter (Enquist and Leimar 1987). However, individual participation may be negatively correlated with the size of one’s own group (Olson 1965; Esteban and Ray 2001; Pride et al. 2006)—perhaps because of greater opportunities for free-riding (Nunn and Deaner 2004).

We set out to study patterns and determinants of individual variation in the propensity to participate in intergroup conflicts in Verreaux’s sifakas (*Propithecus verreauxi*), a group-living primate from Madagascar. Verreaux’s sifakas are a suitable and interesting species to test factors influencing individual participation in group encounters for several reasons. First, they exhibit interesting territorial behavior, characterized by partial home range overlap with neighboring groups and core areas for exclusive use (Jolly 1966; Benadi et al. 2008). Second, they live in relatively small groups of about six (SD = 2) individuals, which provide an opportunity to study the role of collective action in small groups, where each individual represents a significant proportion of total group size. Third, sifakas lack sexual size dimorphism, and females are dominant over males in dyadic agonistic interactions (Jolly 1966; Richard and Nicoll 1987), offering an opportunity to study differences in male and female participation, regardless of physical superiority of one sex.

In a field study of eight neighboring groups of Verreaux’s sifakas, we tested the prediction that, due to the similar body size of males and females, and the fact that sifaka females are philopatric and dominant over males, both sexes should participate in group defense. We also predicted that in addition to sex, factors such as age, group size, food availability, the duration of an encounter, and the level of aggression among contestants should influence individual participation. However, only males should increase their participation during the annual mating season when potential mating opportunities in neighboring groups are present (females have been reported to mate with non-resident males: Richard 1985). Moreover, we predicted that dominant individuals of both sexes should participate more often in group encounters than subordinates. In females, we expected that the presence of dependent infants would decrease their probability of participating in an encounter. Finally, we explored the circumstances, such as group size and social status, under which individuals from both sexes did not participate in an intergroup conflict.

## Methods

### Study site and species

The study was conducted in Kirindy Forest, a dry-deciduous forest in western Madagascar (44° 39′ E, 20° 03′ S), a field site operated by the German Primate Center (Kappeler and Fichtel 2012) and situated within a forestry concession managed by the Centre National de Formation, d’Etudes et de Recherche en Environment et Forèstiere (CNFEREF). The regional climate is characterized by pronounced seasonality, with a long dry season from April to November, and a short wet season between December and March. As part of an ongoing long-term project, animals are habituated and individually marked with combinations of colored nylon collars and pendants or color-coded radio collars (Kappeler and Fichtel 2012).

Two observers (FK and M. Razafindrasamba, a Malagasy field assistant, who has conducted behavioral observations of sifakas for more than 13 years) conducted 1-h continuous focal observations (Altmann 1974) on the adults in two different groups simultaneously, resulting in 1480 h of observations distributed between March 2012 and April 2013. The size of the eight study groups ranged between three and eight individuals (Table 1), with one adult female and one to three adult males per group, with the exception of one group, in which two adult females were present. During the study period, eight out of nine females gave birth. Infants were considered dependent until they reached the age of three months, and individuals between 3 months and 4.5 years were defined as juveniles (Kappeler and Fichtel 2012).

**Table 1:** Variation in group size and composition between the eight study groups from March 2012 to April 2013

Group	Range group size
C	3–5 (1 adult female, 1 juvenile female, 1–3 adult males)
E	5–8 (1 adult female, 1 juvenile female, 1–3 adult males, 1–3 juvenile males)
F	4–6 (1 adult female, 1–2 juvenile females, 2–3 adult males)
F1	5 (1 adult female, 1 juvenile female, 2 adult males, 1 juvenile male)
G	4–5 (1 adult female, 1 juvenile female, 1–4 adult males)
H	3–4 (1 adult female, 1–2 adult males, 1 juvenile male)
J	6–8 (2 adult females, 1–2 juvenile females, 3–4 males)
L	3–5 (1–2 females, 2–3 males)

### Group encounters

Intergroup encounters were operationally defined as follows: a conflict began when the nearest members of two groups were at a distance of 50 m or less from each other, and it ended when they were again at a distance of more than 50 m for at least an hour. These two criteria were established empirically during a pilot study. Details of group encounters were recorded with a digital voice recorder (Olympus WS 650S) and subsequently transcribed. The following details on group encounters were recorded: date, time the encounter started and ended, presence of dependent infants, and identity of individuals participating actively in the encounter from the focal and opponent group. Active participation was considered when individuals showed one of the following behaviors during the encounter: scent-marking, vocalizing, chasing, or physical aggression towards a member of another group.

We distinguished four levels of aggression: (1) there was no physical interaction and no vocalization, (2) the interaction between the groups was exclusively vocal and at least one individual produced loud calls, i.e., “tchi-faks” (Fichtel and Kappeler 2002), (3) at least one individual from one group chased one or several members of the other group, and (4) at least one individual from one group displayed physical aggression towards a member of the other group, such as chasing, grappling or biting an opponent. We used two different approaches to infer group size, the total group size referring to the total number of individuals in the group, and the effective group size referring to the number of individuals actively participating in the conflict (i.e., total group size minus non-participating individuals).

### Food availability

We registered the monthly phenology of 693 potential feeding trees distributed throughout the home ranges of the study groups, representing 163 species from 44 families. A semi-quantitative method to infer food availability by assigning scores ranging from 0 to 4 for availability of each item (young leaves, mature leaves, fruits, flowers), with 0 representing total absence of the item, and 4 representing 100 % availability of the item (Fournier 1974). We averaged the scores of food availability for each month and item (young leaves, mature leaves, fruits, and flowers), and also across items to obtain a total score for monthly food availability. For the statistic analyses, we included only sifakas feeding trees.

### Statistical analyses

All analyses were conducted in R (R, version 3.1.2; R Development Core Team, 2014) and were based on data from the perspective of the focal group. To compare the rate of encounters per month between the dry and the wet season, we used a Generalized Linear Model with the rate of encounters as the dependent variable and season (dry or wet) as the independent variable. We used binomial generalized linear mixed models (GLMM; Baayen et al. 2008), from the package lmer4 (Bates et al. 2014), to investigate whether individual participation (yes or no) was influenced by sex, age, food availability, duration of the encounter, level of aggression (ranging from 1 to 4), and different measures of resource-holding potential of groups, i.e., total and effective group size. Because the duration of encounters was correlated with the effective size of the opponent group (Spearmen rank correlation: *r*_s_ = 0.33, *P* < 0.001), and the level of aggression was also correlated with the effective size of the opponent group (Spearmen rank correlation: *r*_s_ = 0.49, *P* < 0.001), we tested these variables in separate models and used Akaike information criterion (AIC) values for model selection (Bolker et al. 2008). We additionally tested several models including the different variables related to group size as predictors of individual participation in the focal group, using AIC values for model selection. These variables included total size of the focal and opponent group, the difference in total group size, and the effective size of the opponent group as well as the difference in effective group size of the focal and opponent group. The final model included individual participation (yes or no) as response and sex, age, food availability, total group size of the focal group, and the effective group size of the opponent group as predictors and group ID nested in group dyad ID as random factors. However, due to the theoretical relevance of the influence of the total group size of the opponent group, we present and discuss the results of both models.

In order to investigate factors that influence female participation, we used a binomial GLMM, with participation as response variable and the presence of dependent infant as explanatory variable, controlled by individual identity nested in dyad identity. To investigate factors that influence male participation, we used a binomial GLMM, with participation as response variable and social status and mating season (yes or no) as explanatory variables, also controlling for individual identity nested in group dyad identity. Social status was included as an explanatory variable only for male participation because seven out of eight groups harbored only one adult female. Social status of males was based on the following classification (Kappeler et al. 2009): dominant (D): a male that is not related to the dominant female, has higher access to the female(s), and is likely to sire the majority of offspring in the group (Kappeler and Schäffler 2008); natal subordinates (NS): presumably the offspring of their groups’ females; non-natal subordinates (NNS): males that immigrated into the group and are neither related to the dominant female nor to the dominant male; and related (R): males that are related to the dominant male but not to the resident females.

For all models, we checked the relevant assumptions and verified and presented the significance of the full model (including the predictors and control factors) to the null model (only with the control factors), using the R function ANOVA. It was not possible to record data blind because our study involved focal animals in the field.

## Results

### General characteristics of intergroup encounters

**Fig. 1:**
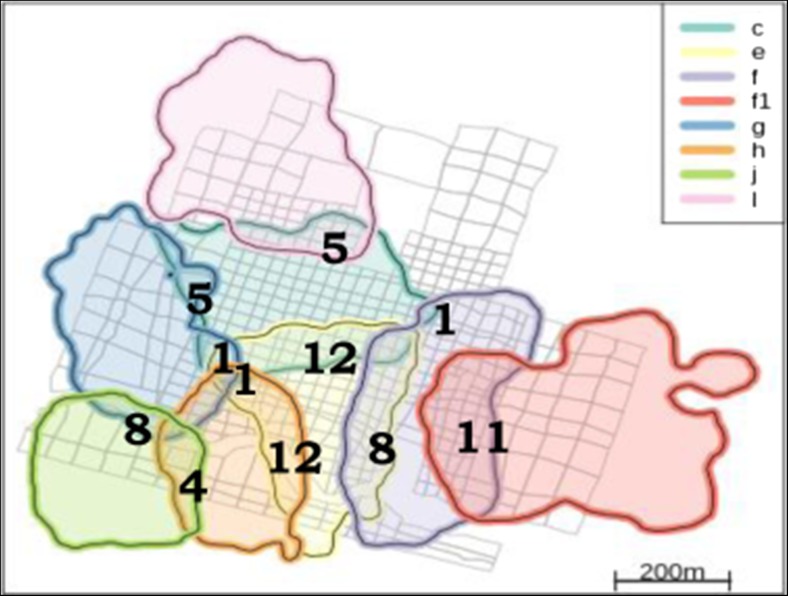
Home ranges of the eight groups of sifakas; annual overlap areas between neighboring groups are based on 95 % kernels. The *numbers* represent the number of observed encounters between each dyad

We observed 88 direct encounters between neighboring groups of sifakas. However, all the following results are based on the analyses of 71 encounters because only they involved two of our study groups (Fig. 1). On average (±SD), sifakas had 6 ± 3 encounters per month.

The encounters lasted on average for 23 ± 22 min, and the majority of them (72 %) reached aggression level 3, including chases between members of both groups. On average, 64 ± 48 % of the adult females and 71 ± 45 % of the adult males present participated in intergroup encounters, indicating that individuals of both sexes regularly did not participate. Overall, non-participation by at least one adult individual occurred in 72 % of intergroup encounters. The frequency of encounters did not differ between the wet and the dry season (*χ*^2^ = 0.47, *df* = 1, *P* = 0.49, Table 2).

**Table 2:** Results of the GLM testing seasonal differences in encounter rate

Seasonal differences in encounters				
Fixed effects	Estimate	SE	*Z* value	Pr(>|z|)
Intercept	1.92	0.13	14.30	0.001
Wet season	−0.42	0.27	−1.61	0.12

### Participation in intergroup conflicts

On the individual level, females participated as often as males and adult individuals participated in encounters more often than juveniles (*χ*^2^ = 33.57, *df* = 3, *P* < 0.001, Table 3). Participation of both, females and males was positively influenced by the effective size of the opponent group, indicating that individuals were more likely to participate when more members of the opponent group participated actively in an encounter. Interestingly, the probability of individual participation was lower in larger groups, i.e., the number of non-participants increased with group size (Table 3). Food availability did not influence individual participation (Table 3). By running the model with the same variables, but exchanging the effective group size with the total group size of the opponent group, there was no influence of total group size on individual participation (*P* = 0.82, Table 4), suggesting that individuals based their decision to participate on how many individuals from the opponent group actively participated. Moreover, the duration of encounters correlated positively with the effective size of the opponent group (Spearmen rank correlation: *r*_s_ = 0.33, *P* < 0.001), and the level of aggression was also correlated with the effective size of the opponent group (Spearmen rank correlation: *r*_s_ = 0.49, *P* < 0.001), suggesting that more aggressive encounters lasted longer and included more participants.

**Table 3:** Results of the binomial GLMM testing the influence of sex, age classes (adults and juveniles), food availability, total size of the focal group, and effective size of the opponent group on participation in intergroup encounters

Fixed effects	Estimate	SE	*Z* value	Pr(>|z|)
Intercept	0.89	0.83	1.06	0.28
Sex	0.33	0.37	0.88	0.38
Age (juveniles)	–1.26	0.41	–3.05	0.002**
Food availability	−0.27	0.63	−0.44	0.66
Total size of focal group	−0.24	0.12	−2.10	0.03*
Effective size of opponent group	0.61	0.13	4.58	<0.001***

*<0.05; **<0.01; ***<0.001—significance levels

**Table 4:** Results of the binomial GLMM testing the influence of sex, age classes (adults and juveniles), food availability, total size of the focal group, and total size of the opponent group on participation in intergroup encounters

Fixed effects	Estimate	SE	*Z* value	Pr(>|z|)
Intercept	2.32	0.94	2.48	0.01*
Sex	0.29	0.32	0.89	0.37
Age (juveniles)	–1.04	0.35	–2.98	0.003**
Food availability	–0.20	0.59	–0.35	0.73
Total size of focal group	–0.24	0.10	–2.39	0.02*
Total size of opponent group	–0.03	0.12	–0.22	0.82

*<0.05; **<0.01; ***<0.001—significance levels

**Fig. 2:**
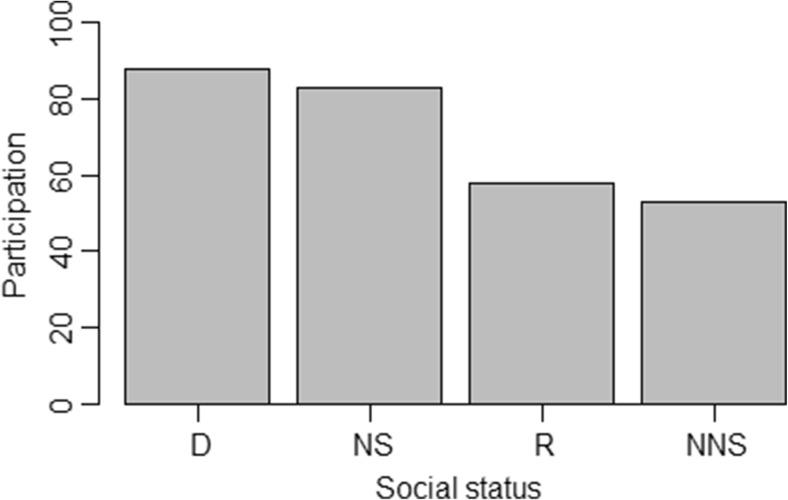
Percentage of male participants in group encounters according to their social status. *D* dominant males, *NS* natal subordinate males, *R* subordinate males that are related to the dominant male, *NNS* non-natal subordinate males

Females participated less often in group encounters when they had dependent infants (*χ*^2^ = 4.42, *df* = 1, *P* = 0.03, GLMM: estimate, 0.85; SE, 0.42, *P* = 0.04). Participation of males was influenced by social status (*χ*^2^ = 18, *df* = 1, *P* = 0.001, Table 5). Dominant males participated in almost all (91 ± 28 %) encounters and did so more often than related and non-natal subordinate males (Fig. 2), suggesting that males with reduced opportunities to reproduce participate less often. Interestingly, participation of males was not affected by mating season.

**Table 5:** Results of the binomial GLMM testing the influence of social status (non-natal subordinate males (NNS), natal subordinate males (NS), subordinate males that are related to the dominant males but not to the group females (R)) and the mating season on the probability of adult males to participate in group encounters

Fixed effects	Estimate	SE	*Z* value	Pr(>|z|)
Intercept	1.91	0.38	4.99	<0.001
Social status				
NS	–0.32	0.74	–0.43	0.66
NNS	–1.94	0.60	–3.27	0.001 **
R	–1.59	0.46	–3.46	<0.001 ***
Mating season	0.27	0.54	0.51	0.61

**<0.01; ***<0.001—significance levels

## Discussion

In this study, we show that both adult female and male Verreaux’s sifakas are regularly involved in intergroup encounters throughout the year. The incentive to participate in intergroup conflicts in both sexes was not influenced by variation in food availability but by group size; in larger groups non-participation occurred more often. In addition, male and female participation were influenced by different factors, i.e, social status and presence of infants, respectively. Moreover, Verreaux’s sifakas seem to base their decision to participate or not in intergroup conflicts also on the circumstance of an encounter, i.e., the number of active opponents. We discuss these incentives and circumstances below and place them in a comparative context.

### Sex and participation in intergroup conflict

Males and females have different incentives for engaging in group defense, and depending on the nature of the encounter, the participation of one sex can be more pronounced than the other. In contrast to other mammals, such as spotted hyenas and lions, in the majority of primates, males participate more often in group encounters than females (reviewed in Table 6). However, in some primates, female participation can be similar or even superior to the participation of males, as for example in blue monkeys (*Cercopithecus mitis*: Cords 2007), ringtailed lemurs (*Lemur catta*: Jolly et al. 1993), or black-tufted marmosets (*Callithrix penicillata*: Decanini and Macedo 2008). The type of social organization (Kappeler and van Schaik 2002), i.e., whether species are organized into multimale and multifemale groups, one male groups or pairs, does not appear to explain sex differences in participation. In Verreaux’s sifakas, females and males participated equally often in group encounters. A combination of factors, including male-male competition over mating opportunities, the lack of sexual size dimorphism, and female dominance over males, may contribute to this pattern. Males may theoretically also participate in group encounters in order to defend food resources for females and infants (the hired-gun hypothesis, reviewed in Fashing 2001; Arseneau et al. 2015), but we found no direct evidence supporting this possibility.

**Table 6:** Level of female participation in group encounters in primate species. The type of social organization does not appear to explain sex differences in participation

Participation of females in comparison with males	Primate species	References	Social organization
Higher	*Cercopithecus diana*	Hill (1994)	OM
	*Cercopithecus mitis*	Cords (2007)	OM
	*Lemur catta*	Nunn and Deaner (2004)	MMMF
Similar	*Propithecus verreauxi*	Present study	MMMF
	*Macaca thibetana*	Zhao (1997)	MMMF
	*Callithrix penicillata*	Decanini and Macedo (2008)	PAIR
	*Cercocebus galeritus*	Kinnaird (1992)	MMMF
	*Cercopithecus ascanius*	Brown (2013)	OM
Lower	*Cebus capucinus*	Perry (1996)	MMMF
	*Colobus guereza*	Fashing (2001)	OM
	*Pan troglodytes*	Williams (2004)	MMMF
	*Macaca fuscata*	Majolo et al. (2005)	MMMF
	*Colobus polykomos*	Korstjens et al. (2005)	OM
	*Gorilla beringei*	Robbins and Sawyer (2007)	OM
	*Alouatta pigra*	Van Belle (2015)	MMMF
	*Presbytis* sp.	van Schaik et al. (1992)	OM
	*Hylobates lar*	Bartlett (2003)	MMMF
	*Papio ursinus*	Cowlishaw (1995)	MMMF
	*Presbytis thomasi*	Steenbeek (1999)	OM
	*Hapalemur griseus*	Nievergelt et al. (1998)	PAIR
	*Cercopithecus aethiops*	Cheney (1981)	MMMF
	*Saguinus mystax*	Garber et al. (1993)	MMMF
	*Cebus olivaceus*	Robinson (1988)	MMMF
	*Macaca maurus*	Okamoto and Matsumura (2002)	MMMF
	*Macaca sylvanus*	Mehlman and Parkhill (1988)	MMMF
	*Macaca radiata*	Cooper et al. (2004)	MMMF
	*Sapajus nigritus*	Scarry (2013)	MMMF
	*Chiropotes sagulatus*	Shaffer (2013)	MMMF
	*Colobus vellerosus*	Sicotte and Macintosh (2004)	OM
	*Pithecia pithecia*	Thompson et al. (2012)	PAIR
	*Lophocebus albigena*	Brown (2013)	MMMF
	*Papio cynocephalus*	Markham et al. (2012)	MMMF

Social organization: one adult male per group (OM), multimale and multifemale groups (MMMF), and one adult male and one adult female (PAIR)

The common pattern of higher male participation observed in primates is in line with the mate defense hypothesis, which postulates that the main incentive for male participation in intergroup encounters is either to defend group females or to obtain access to females of other groups (Wrangham 1980; van Schaik et al. 1992). According to this hypothesis, dominant males in some species achieve higher reproductive success and participate more often in intergroup conflicts than subordinate males (Perry 1996; Gese 2001; Watts and Mitani 2001; Kitchen et al. 2004; Cooper et al. 2004; Van Belle et al. 2014; Arseneau et al. 2015). Particularly during the mating season, dominant males are expected to invest more in intergroup conflicts to maintain access to females and to prevent extra-group copulations. In two populations of Japanese macaques (*Macaca fuscata*), males behaved more aggressively during the mating season in the population in which intergroup mating occurred. Therefore, defending female mates in the mating season was more beneficial in this population than in another one in which extra-group copulations were not observed (Saito et al. 1998).

In our study, dominant males participated more often than subordinate males throughout the year, and the probability of participation of males was not affected by the mating season. The mating season in Verreaux’s sifakas is relatively short, concentrated on 2 months in which females are receptive for a few days (Brockman 1999; Mass et al. 2009). Dominant males mate-guard females during this time, peaking during the short period when females are receptive (Mass et al. 2009). Male reproductive success in sifakas is highly skewed in favor of the dominant male (Kappeler and Schäffler 2008), which indicates that mate-guarding is an effective strategy for dominant males. Moreover, the asynchronous estrus of females within groups allows dominant males to monopolize reproduction in their own groups, resulting in high reproductive skew in favor of the dominant males (Kappeler and Schäffler 2008).

Because rates of extra-group paternities are very low in our study population (Kappeler and Schäffler 2008), dominant males are apparently guarding females of their own group effectively and seem to forgo potential mating opportunities during intergroup encounters, which bear the risk of leaving group females unguarded. Similarly, in banded mongooses (*Mungos mungo*), dominant males guard the breeding females in their own group instead of actively chasing intruders (Cant et al. 2002). Dominant male sifakas might benefit by directing aggression at other groups at all times throughout the year because this might discourage take-over attempts by extra-group males. Similarly, in meerkats (*Suricata suricatta*), where male reproductive success is also highly skewed, dominant males often participate in encounters to keep prospecting males from other groups away. Because outside males sometimes try to take over groups (Port et al. 2011), this could help dominant males to maintain their mating access to females in their own groups.

Female participation in contests between groups is expected when food resources are economically defendable (Wrangham 1980). Food is the limiting factor for female fitness (Trivers 1972), and access to high-quality food can influence the chances of producing more viable offspring, whereas poor nutrition can induce females to delay or skip reproduction, or to compromise infant survival (Bercovitch 1987; Richard et al. 2000; Lewis and Kappeler 2005a; McCabe and Fedigan 2007). In Verreaux’s sifakas, however, food availability did not influence participation in intergroup encounters. Also, the rate of encounters did not differ between the wet and dry season, when food is more or less available, respectively. Since Madagascar’s ecosystems are characterized by pronounced seasonality, coupled with strong climatic unpredictability (Dewar and Richard 2007), and relatively low fruit productivity and nutritional content (Ganzhorn et al. 2009), both sexes may invest equally in resource defense. Furthermore, it has been suggested that female sifakas can be considered capital breeders (Richard et al. 2000; but see also, Lewis and Kappeler 2005b), and are, therefore, expected to compete continuously for food in order to survive and to store nutrients (Richard et al. 2000).

### Intersexual dimorphism and dominance

In the majority of mammals, males are larger than females (Ralls 1976). Since group encounters are physically demanding, being the less-powerful sex can increase the risks and costs of injuries for females, decreasing their motivation to participate in group defense. Accordingly, females in species with pronounced male-biased sexual dimorphism are rarely engaged in group defense (Cheney 1981). Moreover, in species in which males are dominant over females, the lack of dominance can also result in reduced access to the benefits of the conflict, presumably decreasing the motivation of females to join group encounters even more (Cheney 1981; but see, Hill 1994; Cords 2007). In baboons (*Papio cynocephalus*) and white-faced capuchins (*Cebus capucinus*), for example, females are much smaller and socially subordinate to males, and they normally do not contribute to group defense, presumably because of their limited physical power and the skewed access to benefits resulting from the conflict (Perry 1996; Crofoot 2007; Markham et al. 2012). However, because sifakas lack sexual size dimorphism (Kappeler 1991), males and females have similar physical power and possibilities to contribute to group defense. This effect can be crucial in relatively small groups, where each adult represents a relatively large proportion of the group.

Furthermore, because of female dominance, females may have more incentives to participate in group encounters because they may expect or obtain a larger portion of the direct benefits (Cheney 1987; Cords 2007; Kappeler et al. 2009; Van Belle et al. 2014). Female dominance over males is considered an adaptive behavioral mechanism that provides adult females with feeding priority, which is thought to be beneficial or even required under the energetic stress of reproduction for females (Jolly 1984; Young et al. 1990; Wright 1999). Females in ringtailed lemurs, which are also dominant over males, participate regularly and even more often than males in intergroup conflicts (Jolly et al. 1993; Nakamichi and Koyama 1997; Nunn and Deaner 2004).

### Circumstances of intergroup conflict

Despite the difference in incentives for males and females in participating in intergroup encounters, the particular circumstances of each encounter, such as the size of the groups involved, can play an important role in the individual decision of joining encounters. In both sexes, participation in encounters was influenced by the effective size of the opponent group. The ability to assess the number of individuals in the opponent group has been observed in several other species of mammals, such as black howler monkeys (*Alouatta pigra*: Kitchen 2004, 2006), chimpanzees (Wilson et al. 2001), lions (McComb et al. 1994), and spotted hyenas (*Crocuta crocuta*: Benson-Amram et al. 2011).

The number of active individuals in the opponent group may serve as an estimate of the power of the opponent group and the risks of the encounter (Arnott and Elwood 2008). The variance in the number and identity of participants of each encounter creates unpredictability and more challenges for the groups to assess the power of their opponents, especially in species with high fission-fusion dynamics in which the total size of parties varies from encounter to encounter. For Taî chimpanzees (*Pan troglodytes verus*), for example, it has been suggested that this uncertainty allows small parties to attack much larger ones (Boesch et al. 2008). During our study, group size varied between three and eight individuals. Although neighboring groups differed in total group size between 0 and 130 %, absolute group size had no effect on the outcome of intergroup conflicts. Thus, the unpredictability of who will participate in intergroup encounters suggests that Verreaux’s sifakas do not benefit per se from living in larger groups (Kappeler et al. 2009; Port et al. 2011).

In conflicts between single individuals, the duration of encounters increases as the asymmetries in contestant’s power decrease, and therefore the outcome takes longer to be decided (Enquist and Leimar 1983). In this context, in disputes between groups, it is expected that individuals may decide to participate during an ongoing encounter in order to counterbalance the asymmetry to facilitate a favorable outcome. In sifakas, the effective group size of the opponent group was positively correlated with the level of aggression and with the duration of encounter. The fact that longer and more aggressive encounters reached the higher level of participation may suggest that individuals decided to join during the ongoing encounter in accordance to its level of severity. Hence, detailed information on the timing and order of individual participation is required in future research to confirm this assumption. In fact, studies on collective action discussed cooperation among group-living animals as being a simultaneous process, i.e., all group members decide to participate in the encounter at the same time. In that sense, the possibility that an individual could decide to join an ongoing encounter is not considered, dismissing the different timing of actions between individuals in the same social group (Gavrilets 2015). However, it is likely that decisions in cooperating in collective actions are part of an ongoing and dynamic process, where individuals decide their contribution along ongoing collective actions (Hardin 1982).

### Collective action problems in small groups

Collective action problems (CAP; Olson 1965) occur whenever collective action creates a public good (such as a territory) and the selfish interests of group members are not in line. Natural selection will favor free-riders over cooperators, as they reap the benefits of access to the good without risking the costs of producing it (Nunn 2000). In the context of territorial defense, some individuals can be less cooperative than others and still get their share from the benefits of collective actions, thereby undermining group-level cooperation (Nunn 2000; Nunn and Deaner 2004). CAPs in territorial defense have been reported in several mammals (Heinsohn and Packer 1995; Gese 2001; Bonanni et al. 2010) and appear to be common in primates (van Schaik 1996; Nunn 2000; Kitchen and Beehner 2007; Willems et al. 2013; Willems and van Schaik 2015), where they occur in the context of territorial advertisement (Kitchen 2004; Van Belle et al. 2014; Van Belle 2015) and actual intergroup conflicts (Nunn and Deaner 2004; Harris 2010; Crofoot and Gilby 2012). Comparative analyses across primates also indicated that CAPs are less likely to occur in species that are either cooperative breeders, in which the dominant sex is philopatric or that live in relatively small groups with only few individuals of the dominant sex (Willems et al. 2013; Willems and van Schaik 2015). Although Verreaux’s sifakas live in relatively small groups with only few individuals of the dominant sex, and females are philopatric, non-participation of adult individuals occurred regularly, i.e., in 72 % of observed encounters, and was more common in larger groups.

Which factors may explain non-participation in intergroup encounters in sifakas? Infanticide avoidance has been suggested to explain female non-participation in intergroup defense in blue monkeys (Cords 2007), white-faced capuchins (Crofoot and Gilby 2012), and ringtailed lemurs (Nunn and Deaner 2004). Similarly, sifaka females did not participate in intergroup defense when they had dependent infants. Encounters are highly aggressive events in which infants might be harmed and exposed to infanticidal males (van Schaik 1996). Infanticide has been reported several times in *Propithecus* (Erhart and Overdorff 1998; Morelli et al. 2009), including our study site (Lewis et al. 2003; Kappeler and Fichtel 2012). Moreover, sifakas lactate during the peak dry season, when the availability of food is low, so that energetic constraints resulting from the costs of lactation may restrict female participation in group encounters (Harrison 1983).

Dominance status influenced participation in several species because dominant individuals have priority of access to resources (Cheney 1981; Watts and Mitani 2001; Cooper et al. 2004; Kitchen et al. 2004; Van Belle et al. 2014). In sifakas, male non-participation was influenced by social status, with males having fewer opportunities to reproduce participating less often. This is in line with other studies showing that subordinate males participated less often than dominants in the context of group defense (Perry 1996; Gese 2001; Cooper et al. 2004; Kitchen et al. 2004; Crofoot and Gilby 2012; Van Belle et al. 2014; but see, Scarry 2013). Occasional participation of subordinate males might be due to the fact that they may try to avoid potential costs of losing an intergroup encounter. For example, in white-faced capuchins, loosing groups traveled over longer distances than the winning group (Crofoot 2013). Hence, all group members have to pay these costs and, therefore, subordinate individuals might not share the same benefits of winning the encounter as the dominants, but they will certainly pay for at least some of the costs of losing it. In Verreaux’s sifakas, it is puzzling why these subordinate non-natal males are tolerated in the group: on the one hand, they fight with dominant males over access to females in the mating season, but, on the other hand, they do not provide any long-term benefit in terms of infant survival, take-over risks by strange males, or territorial defense (Kappeler et al. 2009; Port et al. 2012). Despite the fact that there is a chance that subordinate non-natal males can impact levels of scramble competition over food, it has been suggested that they are not costly in terms of intragroup feeding competition (Kappeler et al. 2009). Therefore, it is likely that dominant males might tolerate their presence because they at least occasionally participate in intergroup aggression.

## Conclusions

In conclusion, the complexity of intergroup relations appears to be the product of the variable circumstances of each encounter, which impact patterns of individual participation in group encounters. In Verreaux’s sifakas, all group members were rarely observed to engage simultaneously in communal range defense. Thus, decisions to join an intergroup conflict are made opportunistically, most likely based on a combination of individuals’ incentives and the actual circumstances of an encounter. The effective, rather than absolute group size may therefore also be a better predictor of individual participation in other taxa.
